# Efficacy and safety of extracorporeal shock wave therapy for upper limb tendonitis: a systematic review and meta-analysis of randomized controlled trials

**DOI:** 10.3389/fmed.2024.1394268

**Published:** 2024-07-30

**Authors:** Yongqing Xiong, Tianshan Wen, Songzhi Jin, Ling Lin, Qianer Shao, Yue Peng, Qining Zheng, Wei Li

**Affiliations:** Gannan Medical University, Ganzhou, Jiangxi, China

**Keywords:** extracorporeal shock wave, upper limb tendonitis, rotator cuff tendonitis, lateral epicondylitis, randomized controlled trials

## Abstract

**Objective:**

This study synthesized the highest level of evidence to analyse the effectiveness and safety of using extracorporeal shock wave therapy (ESWT) to treat upper limb tendonitis, which was unknown.

**Design:**

We conducted a systematic review and meta-analysis of 18 randomized controlled trials (RCTs) in PubMed, Embase, Web of Science, Medline, and the Cochrane Library.

**Methods:**

Two researchers performed the screening, data extraction, literature quality assessment, and heterogeneity analysis of the searched RCTs.

**Results:**

The main types of morbidity included rotator cuff tendonitis, lateral epicondylitis, finger tendonitis, and long bicipital tendonitis. The results of the meta-analysis showed that ESWT was effective in relieving pain in all four types of tendonitis. In addition, ESWT was more effective in relieving pain in patients with upper limb tendonitis than placebo at the 3- and 6-month follow-ups, especially with radial ESWT (RESWT). Data analysis of the forest plot showed that the experimental group with ESWT as an intervention had a significant improvement in function in patients with rotator cuff tendonitis at the 3-month follow-up. However, subgroup analysis showed that low-energy ESWT was effective in improving function in patients with calcified and non-calcified rotator cuff tendonitis, whereas it was not effective in relieving pain.

**Conclusion:**

ESWT can effectively improve the functional activity in patients with rotator cuff tendonitis and may produce positive analgesic effects in patients with upper limb tendonitis. The incidence of adverse effects is low.

**Systematic Review Registration:**

https://www.crd.york.ac.uk/prospero/display_record.php?ID=CRD42023403594, identifier: PROSPERO, CRD42023403594.

## 1 Introduction

Tendonitis is an injurious disorder of tendons that usually shows a specific inflammatory response early in the injury (1–3). Tendonitis often involves the rotator cuff of the upper extremity, long head of the biceps, and wrist extensor tendons (4), which are the preferred sites for tendonitis—especially rotator cuff tendonitis and lateral epicondylitis. As a self-limiting tendonitis, rotator cuff tendonitis and lateral epicondylitis are characterized clinically by pain, swelling, and dysfunction, which severely interferes with activities of daily living. More than 80% of patients with rotator cuff tendonitis develop calcification deposits that result in pain and limited shoulder motion (5), and supraspinatus tendonitis accounts for ~70% of these cases (6). Similarly, the prevalence of epicondylitis is as high as 13.5% (7). Unfortunately, the prevalence of tendonitis is increasing as the population ages, which is not only a financial burden but also a mental burden on the elderly (8).

There are many treatment modalities to treat tendonitis, including non-surgical and surgical approaches. Non-surgical treatment is usually preferred and includes non-steroidal anti-inflammatory drugs (NSAIDs), injection therapy, and centrifugal exercise training. Surgical treatment is considered the last option and is usually chosen after conservative treatment fails (9–11). The use of NSAIDs and injectable corticosteroids are time-sensitive and have proven efficacy in the short-term; however, they could trigger adverse effects (11). Similarly, exercise training, although widely recognized, is not as effective because of the length of the treatment period and the fact that the effectiveness of the treatment is influenced by the degree of damage to the tendon itself (12, 13).

As conservative and surgical treatments for tendonitis are not always successful, new treatments have been developed, including platelet-rich plasma, laser therapy, peloidotherapy and extracorporeal shock wave therapy (ESWT) (14–16). According to previous studies, ESWT for tendonitis is associated with pain relief, tissue repair, and calcific destruction (17, 18). ESWT produces mechanotransduction effects, decreases the concentration of pro-inflammatory factors, activates downstream inhibitory systems, and promotes the associated intracellular chemical reactions and protein synthesis (19–21). Mechanical stimulation reduces the expression of matrix metalloproteinases and interleukins associated with tendonitis (22). ESWT also increases the material conversion rate of the extracellular matrix and induces neovascularization (23), which promotes collagen synthesis for tendon tissue repair. Thus, ESWT has important potential in regenerative medicine as it accelerates inflammation, cellular anabolism, and catabolism to relieve pain and improve function.

ESWT has been widely promoted in clinical practice and is considered the premier conservative treatment for tendonitis; however, the effectiveness of ESWT in treating tendonitis is influenced by energy density, treatment time and lesion location. We collected relevant RCTs for systematic evaluation and meta-analysis, synthesizing the highest level of evidence to analyze the effectiveness and safety of ESWT for treating upper limb tendonitis in terms of pain relief and functional improvement.

## 2 Methods

### 2.1 Protocol and registration

This systematic evaluation and meta-analysis was designed and implemented based on the Preferred Reporting Items for Systematic Reviews and Meta-analysis (PRISMA) guideline (24) and has been registered with Prospero (CRD42023403594).

### 2.2 Inclusion and exclusion criteria

The inclusion and exclusion criteria for literature screening were predetermined to enable more rigorous literature screening. Based on the PICOS principles of the systematic review, the inclusion criteria for this study included: (1) Patients clinically diagnosed with upper limb tendonitis (include rotator cuff tendonitis, lateral epicondylitis, trigger finger and so on; (2) the experimental group received ESWT or RESWT; (3) the control group received placebo treatment (sham treatment); (4) the outcome indicators of functional activity and analgesic effects were assessed by relevant scales, such as a visual analog scale (VAS), the Constant Murley Scale (CMS) and grip strength (GS); and (5) the experimental design was a RCT.

The exclusion criteria were as follows: (1) retrospective studies, animal studies, single-case reports, protocols, reviews, meta-analyses, poster presentations, or conference abstracts; (2) interventions other than placebo or sham; (3) full-text content not available; (4) missing or duplicated experimental data; and (5) non-English literature.

### 2.3 Retrieval strategy

Searches were performed in the PubMed, Embase, Web of Science, Medline, and Cochrane Library databases from the initial availability date to December 2023 to identify studies for inclusion in the quantitative analysis. The main search terms for this study were “tendonitis,” “rotator cuff tendonitis,” “lateral epicondylitis,” “trigger finger,” “upper limb tendonitis,” and “extracorporeal shock wave.” In addition, we manually searched for other relevant literature, such as studies included in some systematic reviews and meta-analyses, to broaden the search for eligible studies. For instance, the following search strategy was used for PubMed: lateral epicondylitis OR tennis elbow OR rotator cuff tendonitis OR trigger finger OR upper limb tendonitis OR tendinopathy [MeSH terms] AND extracorporeal shockwave therapy [MeSH terms] OR shock wave therapy OR radial extracorporeal shock wave therapies OR HIFU therapy OR extracorporeal high intensity focused ultrasound therapy OR ESWT OR REWST. All the relevant words were examined in advance using the PubMed Subject Glossary. A similar search strategy was used for the other databases.

### 2.4 Study selection

All studies were imported into the document management system (Endnote x20), and the management function was used to remove duplicate content. Two researchers then read the title and abstract of each study and screened the studies according to the predetermined criteria. For further screening, two researchers downloaded the full texts and read them, removing studies that did not meet our final inclusion and exclusion criteria. If there is a disagreement about the screening process for a particular study, a consensus will be reached through advice provided by the principal investigator.

### 2.5 Data extraction

Two reviewers independently extracted data and items from the literature. During the information extraction process, the two evaluators obtained complete test data by contacting the original authors when they encountered unclear or missing information. If a response was not received after three consecutive emails, the study was defined as having missing data.

The following data were extracted from all eligible studies: (1) General study information: first author of the study, country, year of publication, sample size, age of patients, sex, disease type, and disease duration; (2) Study characteristics: study design, setting, and inclusion and exclusion criteria; (3) Specific therapeutic parameters: pulse count, energy, and sessions; (4) The outcome measures were pain and function scores reported at baseline and 3 and 6 months. When 3-month data were not available, we used the closest data points from 1 to 3 months of follow-up; and (5) Adverse events.

### 2.6 Quality assessment and risk of bias assessment

Two researchers independently used the Cochrane Risk of Bias tool (Review Manager 5.40) to assess the quality of risk of bias in the included studies (including the risk of bias graph and risk of bias summary). The assessment included blinding of participants and personnel, outcome assessments, allocation concealment, random sequence generation, incomplete outcome data, selective reporting, and other biases. Risk of bias was graded as high, low, or unclear (25).

The quality of evidence for the outcome indicators was assessed using the Grading of Recommendations Assessment, Development and Evaluation (GRADE) system. The assessments included limitations, intermittencies, inconsistencies, and imprecision (26). Evidence levels for each element were categorized as “high,” “moderate,” “low,” or “very low,” with the final strength of recommendation as “strong” and “weak” (27).

### 2.7 Statistical analysis

The data extracted from the included studies were analyzed for statistical and analytical purposes. Heterogeneity between studies was statistically analyzed using RevMan 5.40. The size of heterogeneity was expressed as *I*^2^; *I*^2^ values of 25%, 50%, and 75% represented no significant heterogeneity, moderate heterogeneity, and large heterogeneity in the combined results, respectively (28). Subgroup and sensitivity analyses were performed when moderate heterogeneity was observed in combined results. Moreover, when *I*^2^ ≥ 50%, a random-effects model was used, and when *I*^2^ < 50%, a fixed-effects model was used (29). Subgroup and sensitivity analyses were performed when moderate heterogeneity was observed in the combined results, subgroup analysis and sensitivity analysis were performed (28, 30). The Egger, Begg, and funnel plot methods were also used to test for publication bias. The continuity outcomes were calculated and expressed as 95% confidence intervals (CIs) and mean differences (MDs) to express the effect size.

The outcome indicators of function and pain were assessed using relevant scales such as the VAS, CMS, and GS.

## 3 Results

### 3.1 Results of the literature search

**Figure 1:**
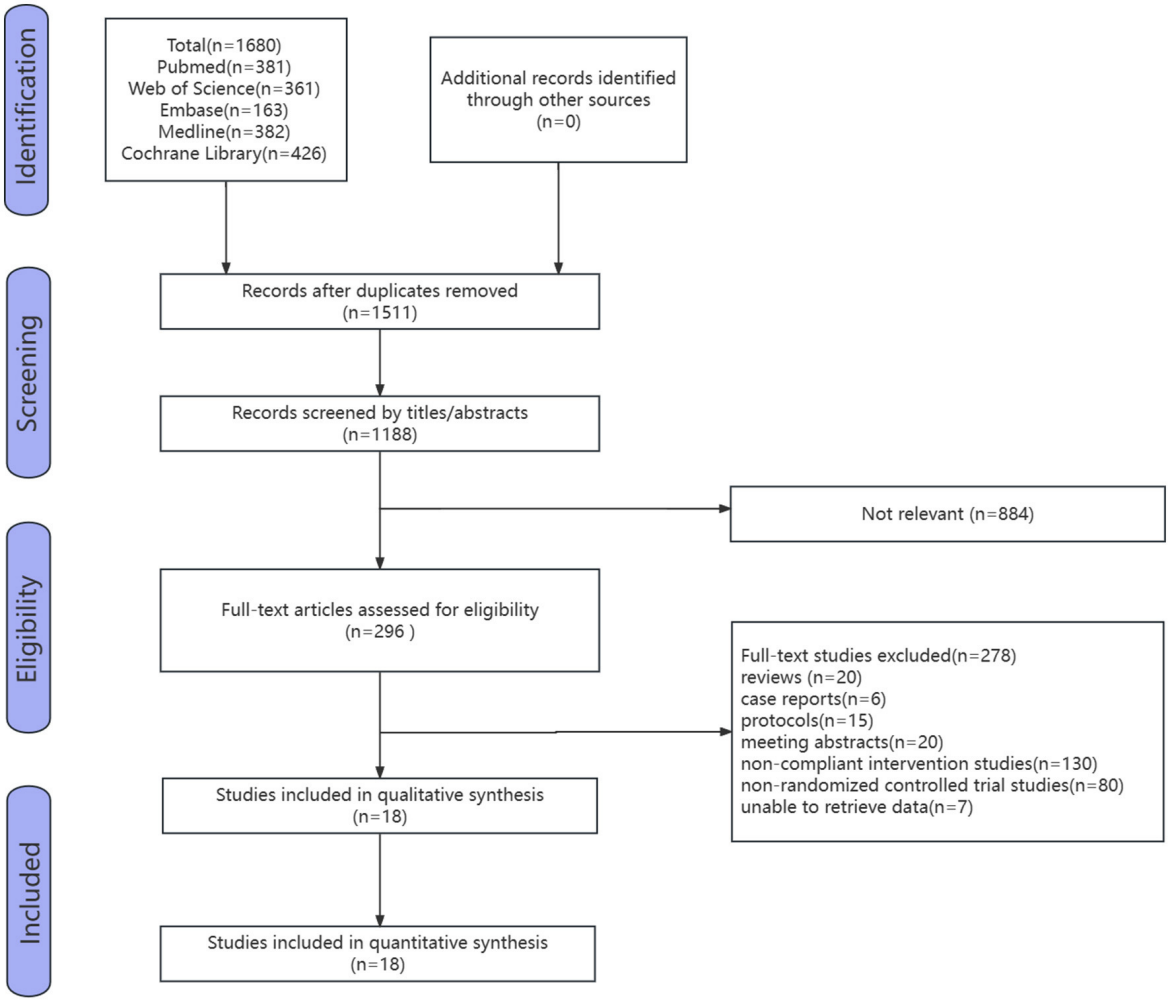
Flow graph of selection and exclusion.

Five databases were searched, and the initial search yielded 1,680 studies. After removing duplicate studies using a literature management software, the results left 1,180 studies. After removing duplicates and selections based on titles and abstracts by the two reviewers, 296 studies were analyzed. After reviewing the full text, 278 studies were excluded, including 20 reviews, six case reports, 15 study protocols, 20 meeting abstracts, 130 non-compliant intervention studies, 80 non-RCTs, and seven studies for which data could not be retrieved. Ultimately, 18 eligible studies were included (Figure 1).

### 3.2 Characteristics of included studies

Basic information and intervention parameters of the 18 RCTs were extracted and are summarized in Table 1. There were eight studies about rotator cuff tendonitis, eight studies about lateral epicondylitis, one study about long bicipital tendonitis, and one study about finger tendonitis. A total of 1,351 patients eligible for the studies were included, with 740 patients treated with ESWT and an additional 611 receiving only placebo as controls.

**Table 1:** Summary of study and patient characteristics.

**References**	**Country**	**Sample size (*n*)**	**Sex (M/F)**	**Age (years)**	**Disease type**	**Disease duration (Mon)**	**Outcomes indicators**	**Adverse events**	**Pulses**	**Energy (mj/mm^2^)**	**Sessions**
Li et al. (17)	China	rESWT:42	–	48.4 ± 9.7	RCT	27.5 ± 11.9	VAS	NO	3,000	0.11	Once every 3 days, 5 sessions
		Placebo:42		46.9 ± 10.1		30.1 ± 12.3	CMS				
Speed et al. (31)	England	ESWT:34	13/21	50.7 ± 11.5	RCT	23 ± 31.0	VAS	Worse symptoms	1,500	0.12	Once a month, 3 sessions
		Placebo:40	18/22	54.2 ± 12.5		23.3 ± 21.0					
Kolk et al. (32)	Netherlands	rESWT:44	12/32	48 ± 9	RCT	24 ± 18	VAS	NO	2,000	0.11	Once every 10–15 days, 3 sessions
		Placebo:38	13/25	46 ± 10.75		29 ± 43.5	CMS				
Gerdesmeyer et al. (33)	Germany	H-ESWT:48	13/35	51.6 ± 8.5	RCT	42.6 ± 23.2	VAS	–	1,500	0.32	Once every 2 weeks, 2 sessions
		L-ESWT:48	16/32	47.3 ± 8.5		42.8 ± 25.2	CMS		6,000	0.08	
		Placebo:48	28/20	52.3 ± 9.8		41.3 ± 28.6					
Galasso et al. (34)	Italy	ESWT:11	7/4	50.7 ± 8.44	RCT	45.36 ± 34.33	CMS	NO	3,000	0.068	Once a week, 2 sessions
		Placebo:9	4/5	51.11 ± 13.26		61.22 ± 24.04					
Loew et al. (35)	Germany	H-ESWT:20	–	–	RCT	–	CMS	NO	2,000	0.3	–
		L-ESWT:20							2,000	0.1	
		Placebo:20									
Schmitt et al. (36)	Germany	ESWT:20	–	–	RCT	22.4 ± 9.7	VAS	–	6,000	0.11	Once a week, 3 sessions
		Placebo:20				18.3 ± 8.3					
Cacchio et al. (37)	Italy	rESWT:45	27/18	56.12 ± 1.98	RCT	14 ± 4.95	VAS	Bloody swelling	2,500	0.10	Once a week, 4 sessions
		Placebo:45	28/17	56.42 ± 2.09		13 ± 5.03					
Capan et al. (38)	Turkey	rESWT:23	7/16	48.4 ± 9.0	LE	7.9 ± 5.2	VAS	NO	2,000	–	Once a week, 3 sessions
		Placebo:22	3/19	46.2 ± 7.4		7.7 ± 5.2	GS				
Pettrone et al. (39)	American	ESWT:56	54/60	47	LE	–	VAS	Pain	2,000	0.06	Once a week, 3 sessions
		Placebo:58					GS				
Rompe et al. (40)	Germany	ESWT:34	18/16	45.9 ± 12.3	LE	23.3 ± 27	VAS	Pain	2,000	0.09	Once a week, 3 sessions
		Placebo:36	18/18	46.2 ± 11.2		25.1 ± 30					
Speed et al. (41)	England	ESWT:40	19/21	46.6 ± 11	LE	15.9 ± 9.75	VAS	Worse Symptoms	1,500	0.18	Once a week, 3 sessions
		Placebo:35	14/21	48.2 ± 8.5		12 ± 9.25					
Yang et al. (42)	China	rESWT:15	8/7	50.93 ± 8.40	LE	6.53 ± 6.45	VAS	NO	2,000	MT	Once a week, 3 sessions
		Placebo:13	4/9	51.08 ± 9.52		7.31 ± 7.61	GS				
Guler et al. (43)	Turkey	ESWT:20	6/14	46.3 ± 8.09	LE	4.1 ± 2.4	VAS	–	1,500	–	1 session
		Placebo:20	6/14	45.8 ± 10.8		4.4 ± 2.2	GS				
Collins et al. (44)	American	ESWT:93	46/47	44 ± 7.61	LE	–	VAS	Pain	–	–	–
		Placebo:90	41/49	46 ± 7.52							
Staples et al. (45)	Australia	ESWT:33	19/14	49.8 ± 7.4	LE	–	VAS	Pain	2,000	MT	Once a week, 3 sessions
		Placebo:30	18/12	49.1 ± 8.8							
Chen et al. (46)	China	H-ESWT:20	5/15	56.2 ± 8.9	TF	–	VAS	NO	1,500	0.01	Once a week, 4 sessions
		L-ESWT:20	6/14	55.6 ± 7.3					1,500	0.006	
		Placebo:20	4/16	54.8 ± 13.4							
Liu et al. (47)	China	Reswt:54	34/20	55.8	LPT	–	VAS	–	1,500	0.12	Once a week, 4 sessions
		Placebo:25	18/7	54.84							

RCT, rotator cuff tendonitis; LE, lateral epicondylitis; TF, trigger finger; LPT, long bicipital tendonitis; NO, no adverse events; “–”, not report; rESWT, radial extracorporeal shock wave therapy; MT, maximum tolerance; VAS, Visual Analog Scale; CMS, Constant Murley Scale; GS, grip strength.

According to the simplest classification about the energy levels of ESWT, with low-energy ESWT having an energy flux density (EFD) of < 0.12 mj/mm^2^ and high energy an EFD between 0.12 and 0.38 mj/mm^2^ (31). Accurate EFD was reported in 13 out of 18 experiments, with five studies for high energy (0.12–0.32 mj/mm^2)^ and 10 studies for low energy (0.01 to 0.12 mj/mm; two of the studies had both high and low-energy groups as well as a placebo group).

### 3.3 Results of the quality assessment

**Figure 2:**
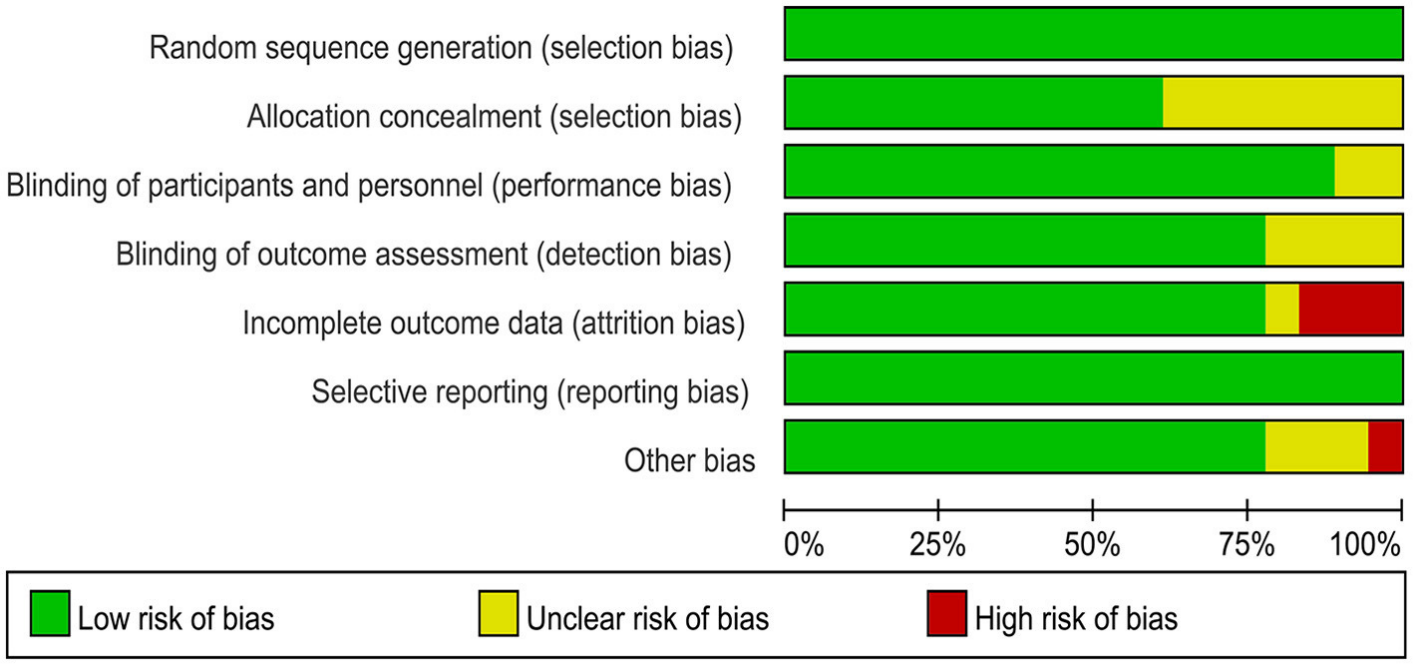
Risk of bias graph.

**Figure 3:**
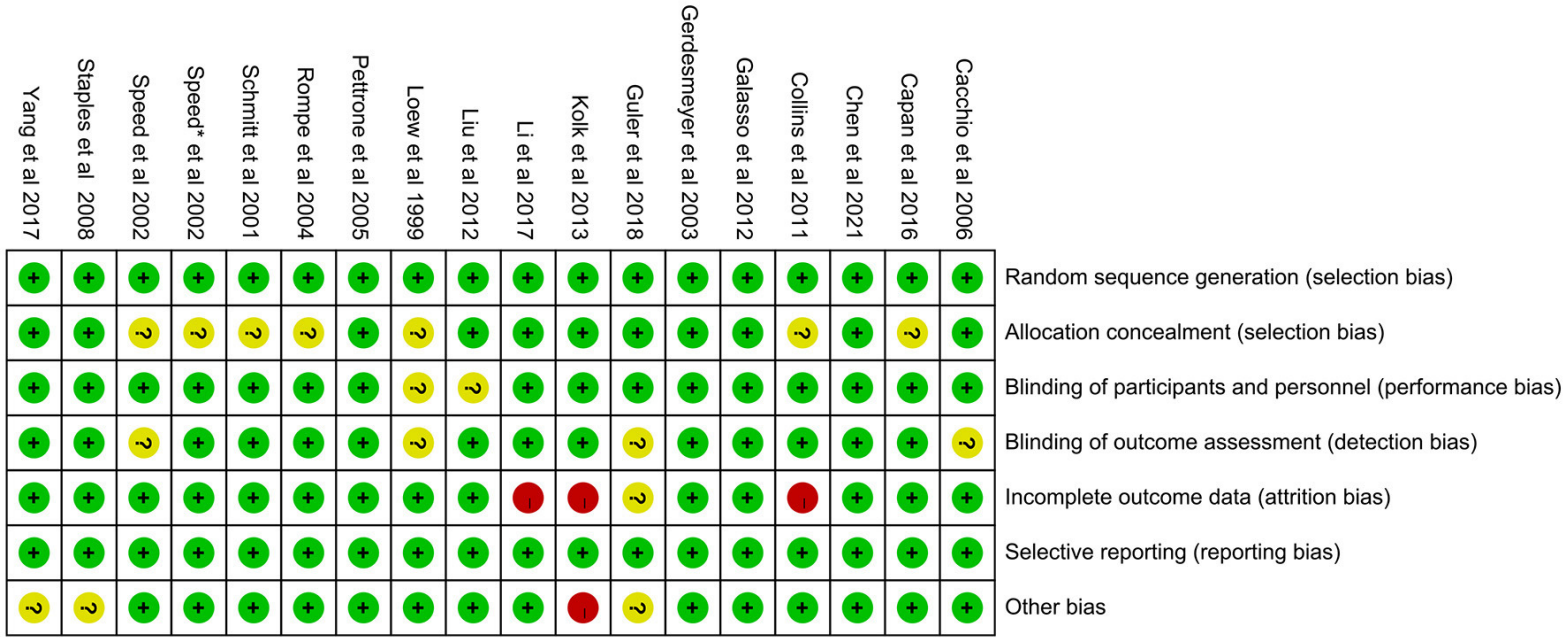
Risk of bias summary.

The 18 included RCTs were assessed using the Cochrane Risk of Bias Assessment Tool (RevMan 5.40) for risk of bias. Three studies had incomplete outcome data, mainly because of a large or unbalanced number of missing people between the groups, and the deficiency of data significantly affected the effect values. In one study, the diagnoses of the included patients were made based on history and physical examination. This could have led to selection bias. Other studies have shown a low or unclear risk in all risk–bias assessments. Overall, the included RCTs had a low risk of bias (Figures 2, 3). We assessed the level of evidence for the main outcome indicators (VAS, CMS, and GS) of the included studies using GRADE. The results of the Egger and Begg tests showed no publication bias in the included studies, according to the three outcome indicators (*P* > 0.05). The quality of evidence for the VAS was moderate, owing to high heterogeneity (*I*^2^ > 80%). Moreover, the quality of evidence for GS was assessed as moderate owing to the smaller sample size and wider CIs. Overall, the GRADE recommendation rating was “strong” for the three outcome indicators (Table 2).

**Table 2:** GRADE evidence profile: ESWT for patients with upper limb tendonitis.

**Outcome**	**Number of studies**	**Design**	**Mean difference (95% Cl)**	**Study limitations**	**Inconsistency**	**Indirectness**	**Imprecision**	**Publication bias**	**GRADE quality**	**Symbolic expression**
VAS	16	RCT	−1.45 (−2.46, −0.45)	0	−1^a^	0	0	0	Moderate	⊕⊕⊕⊖
CMS	6	RCT	7.56 (3.69, 11.43)	0	0	0	0	0	High	⊕⊕⊕⊕
GS	4	RCT	2.38 (−0.43, 5.20)	0	0	0	−1^b^	0	Moderate	⊕⊕⊕⊖

VAS, Visual Analog Scale; CMS, Constant Murley Scale; GS, grip strength.

^a^Heterogeneity is too high (I^2^ > 80%).

^b^Smaller sample size and wider confidence intervals.

### 3.4 Results of pain relief

#### 3.4.1 Short and long-term efficacy

**Figure 4:**
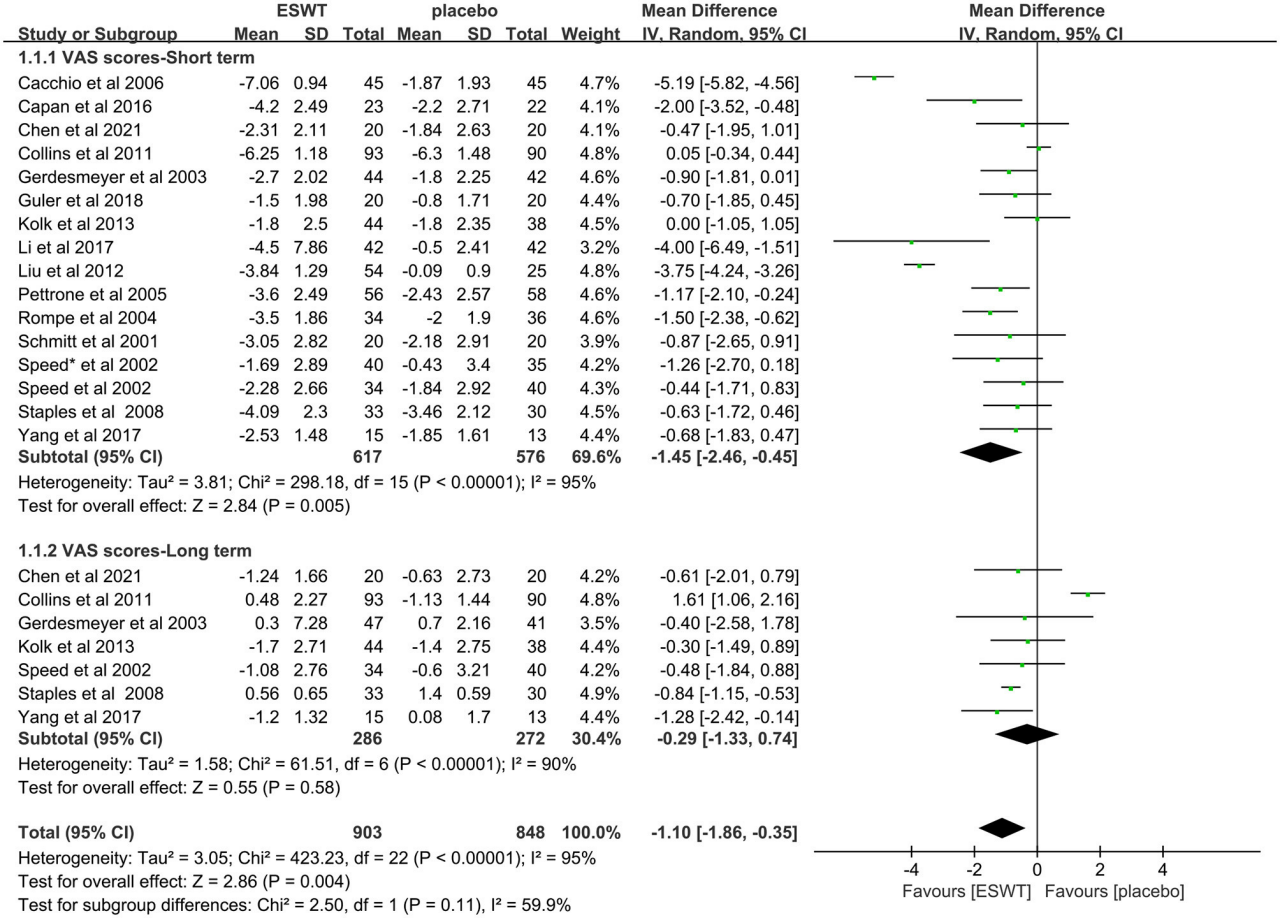
Forest plot for the VAS score.

Sixteen studies reported VAS scores that were used to assess pain intensity (17, 31–34, 36–44, 46, 47). In these studies, 1,751 participants participated in ESWT trials, divided into experimental and control groups. Data analysis of the forest plot showed that the experimental group with ESWT as an intervention had a significant improvement in VAS scores in patients with upper limb tendonitis at both the 3- and 6-month follow-ups [3 months, MD = −1.45, 95% CI: (−2.46, −0.45), *I*^2^ = 95%, *P* < 0.05; 6 months, MD = −0.29, 95% CI: (−1.33, −0.74), *I*^2^ = 0%, *P* = 0.58; Figure 4].

#### 3.4.2 Non-calcific and calcific efficacy

**Figure 5:**
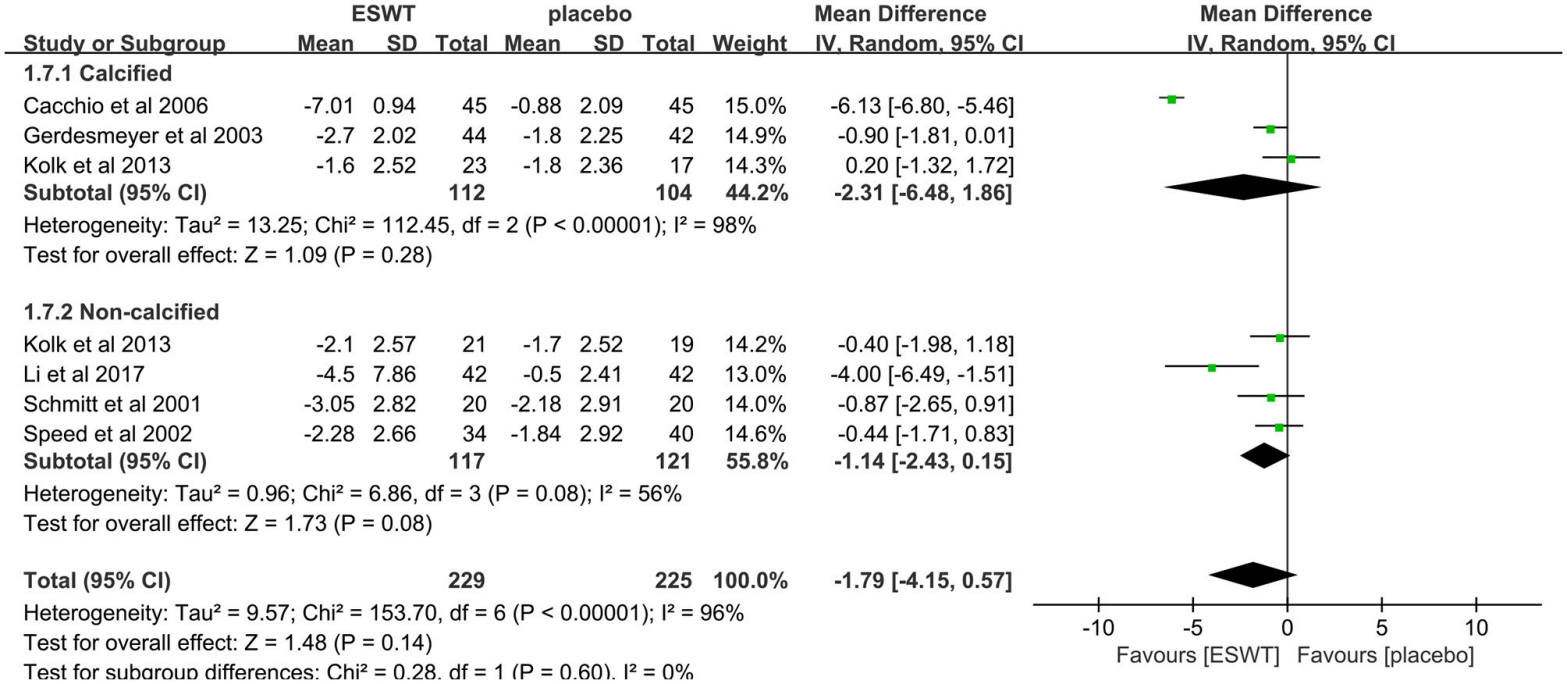
Subgroup analysis for VAS scores with and without calcification.

Five studies compared low-energy ESWT (EFD < 0.12 mj/mm^2^) for calcific rotator cuff tendonitis (32, 33, 37) with non-calcific rotator cuff tendonitis (17, 32, 36). In these studies, 380 participants participated in ESWT trials, divided into experimental and control groups. Subgroup analysis showed that low-energy ESWT was not effective in relieving pain in patients with calcific and non-calcific rotator cuff tendonitis at the 3-month follow-up [calcification, MD = −2.31, 95% CI: (−6.48, 1.86), *I*^2^ = 98%, *P* = 0.28; non-calcification, MD = −1.55, 95% CI: (−3.46, 0.36), *I*^2^ = 67%, *P* = 0.11; Figure 5].

#### 3.4.3 Efficacy of different types of tendonitis

**Figure 6:**
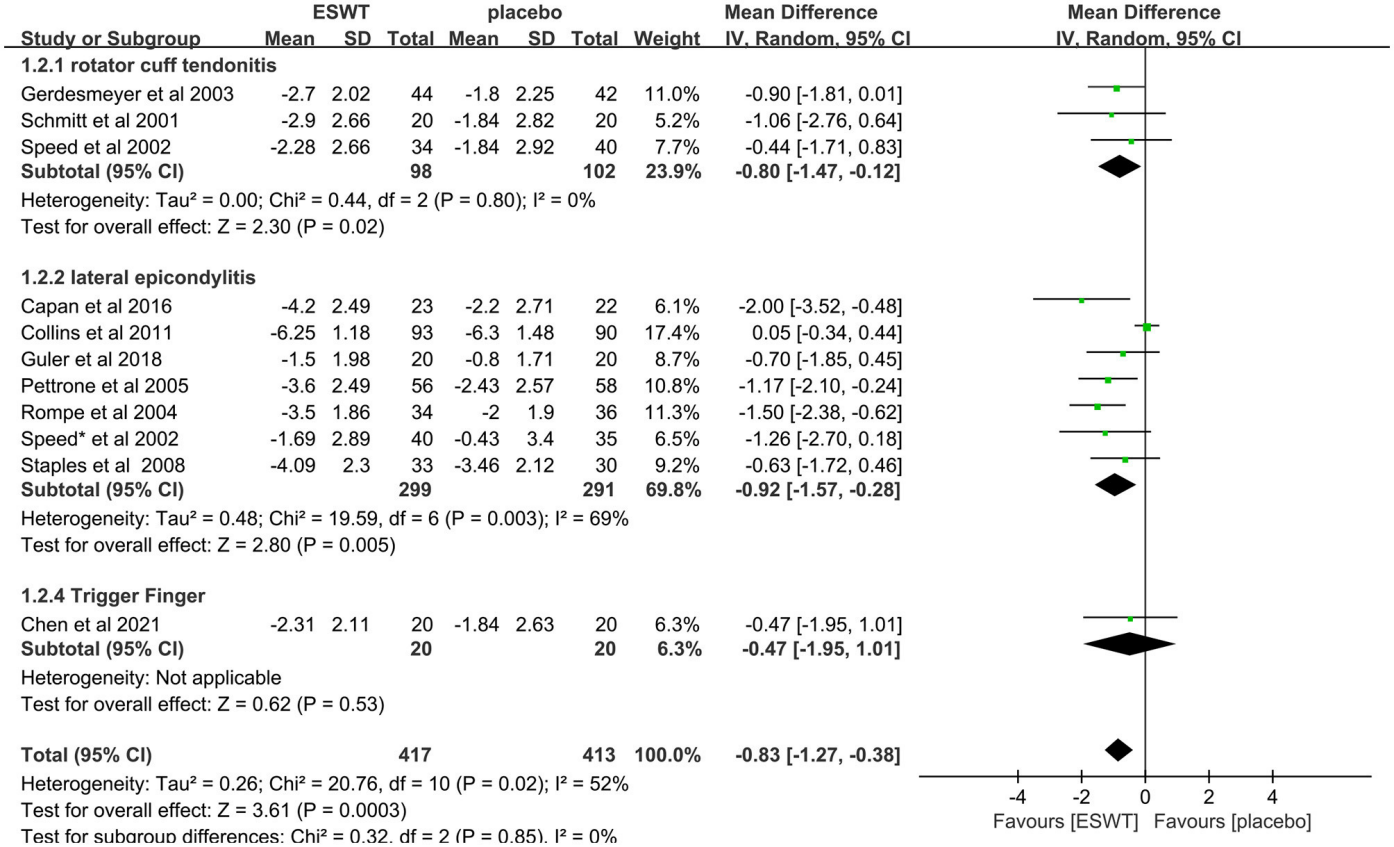
Forest plot for VAS score at different types of tendonitis.

Eleven studies compared ESWT for different types of tendonitis, include rotator cuff tendonitis (31, 36, 46), lateral epicondylitis (38–41, 43–45) and trigger finger (46). In these studies, a total of 830 people were enrolled in the ESWT trial, divided into experimental and control groups, all of whom were treated with ESWT. Subgroup analysis showed that ESWT was effective in reducing VAS scores in patients with tendonitis at the 3-month follow-up [rotator cuff tendonitis, MD = −0.80, 95% CI: (−1.47, −0.12), *I*^2^ = 0%, *P* = 0.02; lateral epicondylitis, MD = −0.92, 95% CI: (−1.57, −0.28), *I*^2^ = 69%, *P* = 0.005; trigger finger, MD = −0.47, 95% CI: (−1.95, 1.281), *P* = 0.53; Figure 6].

#### 3.4.4 ESWT and RESWT efficacy

**Figure 7:**
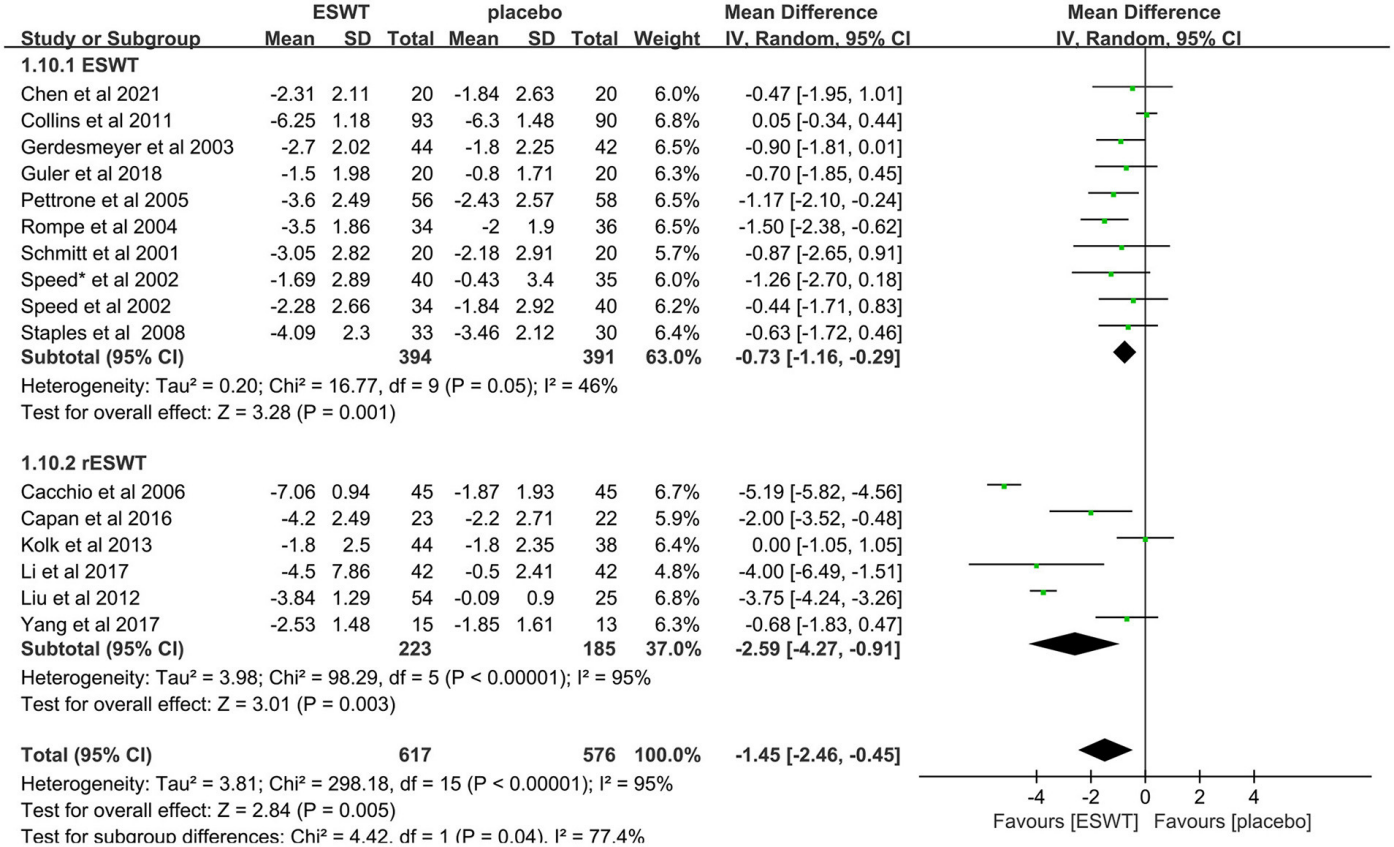
Subgroup analysis for VAS scores at different types of ESWT.

Sixteen studies compared ESWT (31, 33, 36, 39–41, 43–46) and RESWT (17, 32, 37, 38, 42, 47) for the treatment of upper limb tendonitis. In these studies, 1,193 participants participated in ESWT trials, divided into experimental and control groups. Subgroup analysis showed that ESWT and RESWT were both effective in improving pain symptoms in patients with upper limb tendonitis at the 3-month follow-up [ESWT, MD = −0.73, 95% CI: (−1.16, −0.29), *I*^2^ = 46%, *P* = 0.001; RESWT, MD = −2.59, 95% CI: (−4.27, −0.91), *I*^2^ = 95%, *P* < 0.01; Figure 7]. Although there was no significant difference in the efficacy of the two treatments, RESWT was more effective than ESWT in relieving pain [test for subgroup differences, MD = −1.45, 95% CI: (−2.46, −0.45), *I*^2^ = 77.4%, *P* = 0.04; Figure 7]. These results suggest that RESWT should be prioritized in subsequent studies on the treatment of tendonitis.

#### 3.4.5 High- and low-energy efficacy

**Figure 8:**
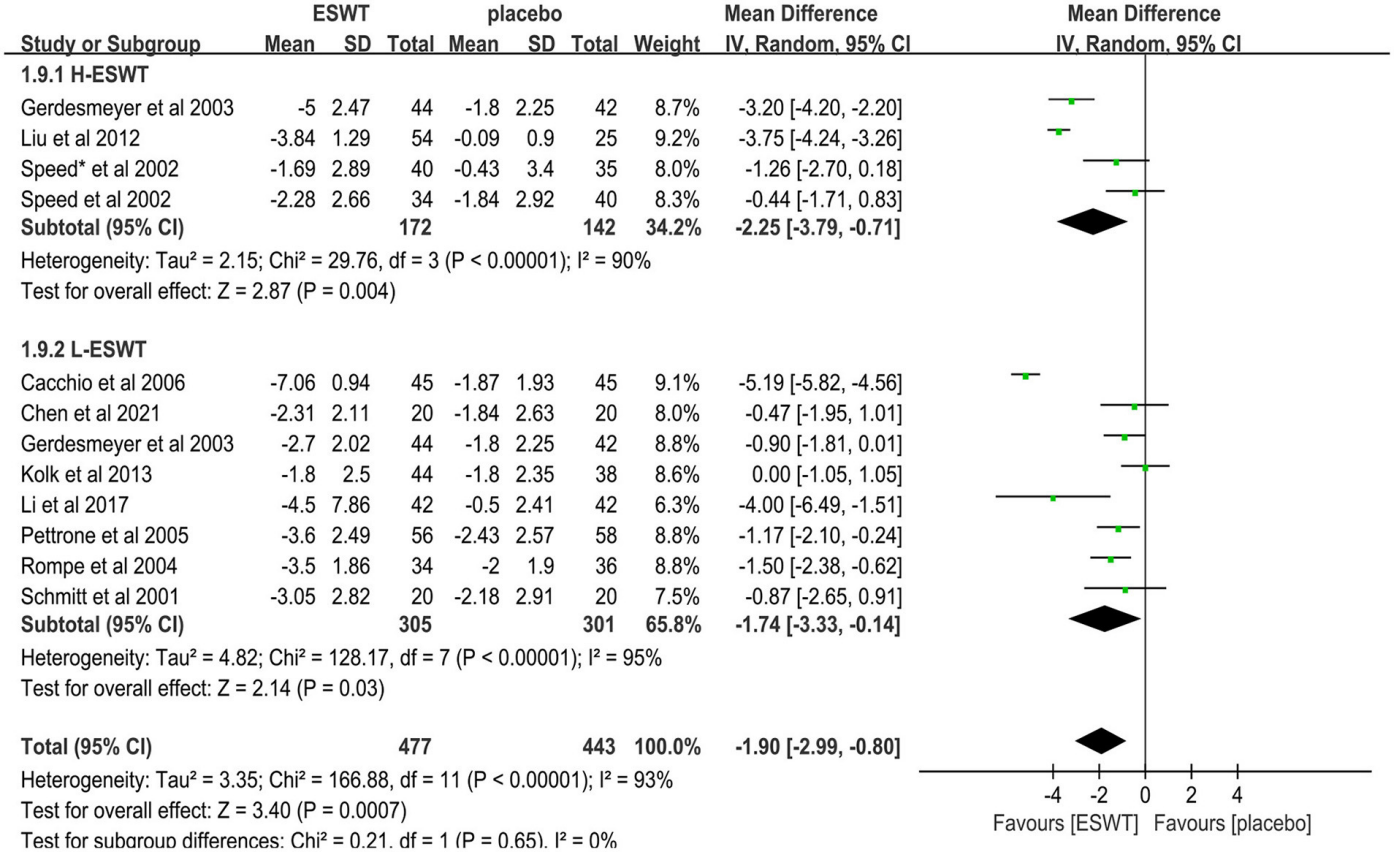
Subgroup analysis for VAS scores at different energy densities.

Eleven studies compared low-energy ESWT (17, 32, 33, 36, 37, 39, 40, 46) and high-energy ESWT (31, 33, 41, 47) for upper limb tendonitis. In these studies, 920 participants participated in ESWT trials, divided into experimental and control groups. Subgroup analysis showed that high-energy ESWT was effective in relieving pain symptoms at the 3-month follow-up [MD = −2.25, 95% CI: (−3.79, −0.71), *I*^2^ = 90%, *P* = 0.004, Figure 8], but the efficacy of low-energy ESWT was not significant compared with the control group [MD = −1.74, 95% CI: (−3.33, −0.14), *I*^2^ = 95%, *P* = 0.03, Figure 8]. In the low-energy group, EFD ranged roughly from 0.01 to 0.12 mj/mm^2^. Therefore, the degree of pain relief was positively correlated with energy density.

### 3.5 Results of functional improvements

**Figure 9:**
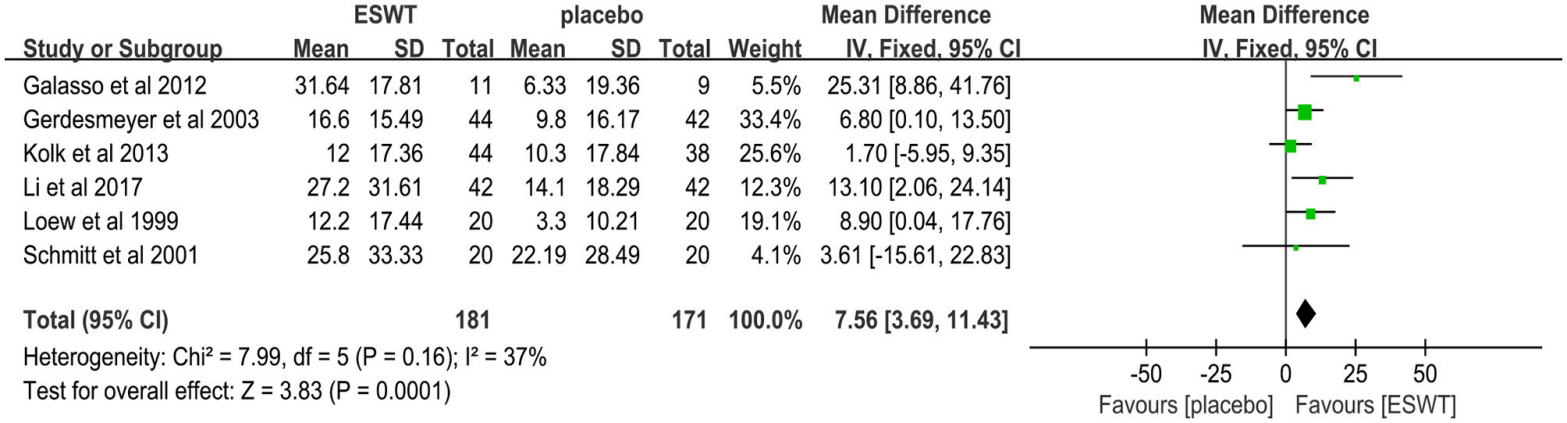
Forest plot for the CMS score.

**Figure 10:**
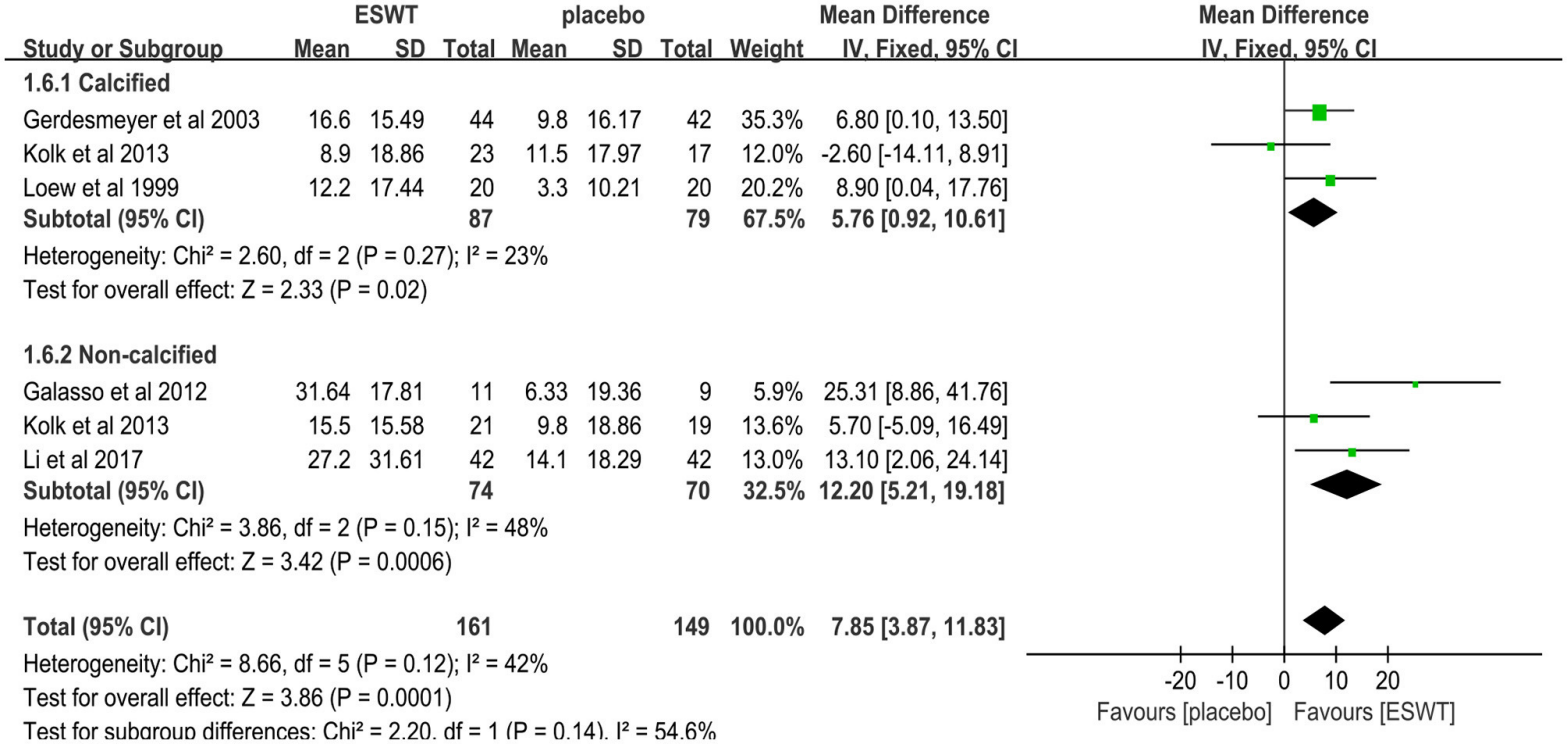
Subgroup analysis for CMS scores with and without calcification.

**Figure 11:**
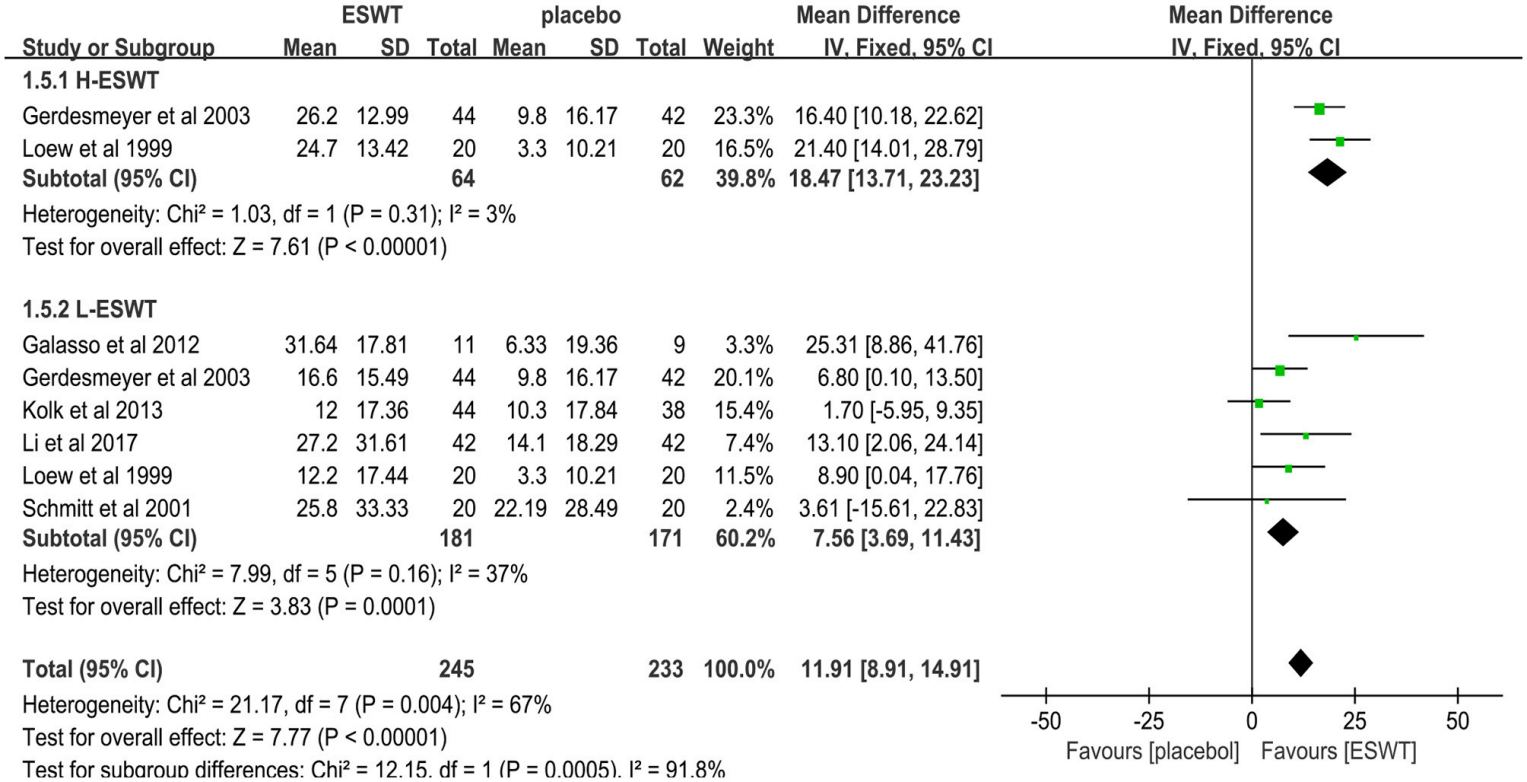
Subgroup analysis for CMS scores at different energy densities.

Six studies reported CMS scores that were used to assess the function intensity involving rotator cuff tendonitis (17, 32–36). In these studies, 352 participants participated in ESWT trials, divided into experimental and control groups. Data analysis of the forest plot showed that the experimental group with ESWT as an intervention had a significant improvement in CMS scores in patients with rotator cuff tendonitis at the 3-month follow-up [MD = 7.56, 95% CI: (3.69, 11.43), *I*^2^ = 37%, *P* < 0.001; Figure 9]. Subgroup analysis based on the presence of calcification showed that ESWT was effective in improving functional activity in patients with calcific and non-calcific tendonitis at the 3-month follow-up [calcification, MD = 5.76, 95% CI: (0.92, 10.61); *I*^2^ = 23%, *P* = 0.02; non- calcification, MD = 12.20, 95% CI: (5.21, 19.18), *I*^2^ = 48%, *P* < 0.001; Figure 10]. Additional subgroup analysis based on the EFD of ESWT showed that ESWT was effective in improving functional activity in patients with rotator cuff tendonitis and suggested that the effect was dose dependent [high, MD = 18.47, 95% CI: (13.71, 23.23); *I*^2^ = 3%, *P* < 0.001; low, MD = 7.56, 95% CI: (3.69, 11.43), *I*^2^ = 37%, *P* < 0.001; test for subgroup differences, MD = 11.91, 95% CI: (8.91, 14.91), *I*^2^ = 91.8%, *P* < 0.001; Figure 11].

**Figure 12:**
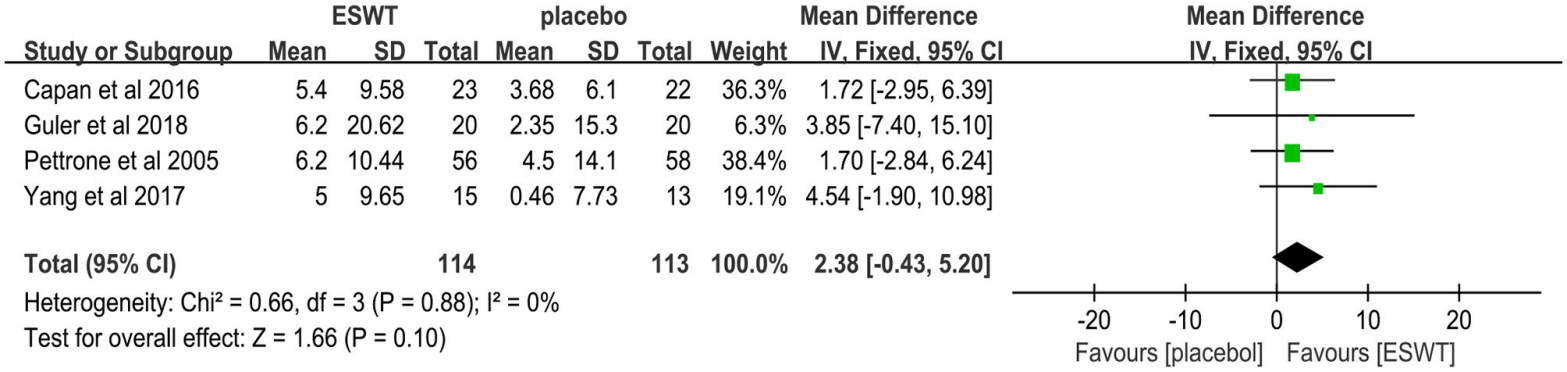
Forest plot for the GS score.

Four studies reported the GS scores that were used to assess power intensity involving lateral epicondylitis (38, 39, 42, 43). In these studies, 227 participants participated in ESWT trials, divided into experimental and control groups. Data analysis of the forest plot showed that the experimental group with ESWT as an intervention had a non-significant improvement in GS scores in patients with lateral epicondylitis at the 3-month follow-up [MD = 2.38, 95% CI: (−0.43, 5.20), *I*^2^ = 0%, *P* = 0.10; Figure 12]. The energy density parameter of ESWT was not mentioned in any of the four studies included in this review. In addition, the evidence level for this outcome indicator was moderate according to the GRADE classification. These findings suggest that additional clinical trials are required to verify the accuracy of our conclusions.

### 3.6 Safety

No serious long-term adverse effects were reported in any of the included studies. Some studies reported a temporary increase in pain and local reactions such as swelling, erythema, petechiae, or small hematomas, but these symptoms disappeared at the end of treatment (33, 34, 37, 39, 44). Only a small percentage of the patients could not tolerate the treatment, had worsening symptoms, or withdrew from the trial (38, 39, 48). Some studies have emphasized the use of local anesthesia or oral analgesia during treatment, which can only be used as a factor to increase heterogeneity without affecting the outcome (33, 36, 44).

## 4 Discussion

The main results of the meta-analysis showed that ESWT was effective in relieving pain and improving function in patients with upper limb tendonitis. In addition, ESWT was more effective in relieving pain in patients with upper limb tendonitis than placebo at the 3- and 6-month follow-up, especially when with radial ESWT. In patients with rotator cuff tendonitis, there was a significant positive improvement in function after 3 months. However, subgroup analysis showed that low-energy ESWT was effective in improving function in patients with calcified and non-calcified rotator cuff tendonitis, whereas was not effective in relieving pain. However, the effectiveness of ESWT for upper limb tendonitis remains influenced by factors such as intervention parameters and differences in the tendonitis type. Therefore, we analyzed ESWT treatment of upper limb tendonitis and discussed the intervention parameters, such as type of ESWT, energy level and Sessions, hoping to provide reference suggestions for the optimal treatment protocol of clinical ESWT treatment.

In recent years, ESWT for rotator cuff tendinitis with or without calcification has received a lot of attention. The selection of ESWT with an appropriate energy density is a prerequisite for the effective treatment of rotator cuff tendinitis. Based on an analysis of previous systematic review, high-energy ESWT is effective in relieving pain, improving function, and effectively dissolving calcifications in rotator cuff tendonitis (49–51). The theory of reactive calcification was proposed by Uhthoff and Loehr (52), who divided calcific tendonitis into three stages: pre-calcification, mid-calcification, and post-calcification. They concluded that in the mid-calcification stage, the calcified tissue wears away from the surrounding normal tissue, indirectly producing an inflammatory response and leading to tissue oedema, which increases patients' pain. ESWT achieves calcification dissolution by producing mechanical stimulation and shattering calcium deposits. However, this effect seems to be influenced by the energy density of ESWT. Low-energy ESWT cannot dissolve all calcium deposits and could leave some behind; the residual calcium deposits continue to stimulate the body to produce an inflammatory response, triggering pain in the patient (53).

Interestingly, combined with the conclusions we obtained, the use of ESWT with EFD < 0.12 mj/mm2 was effective in treating rotator cuff tendonitis. In addition, low-energy ESWT only improves function in patients with calcific tendonitis, and pain outcomes are uncertain (49). Similarly, there is moderate evidence that low-energy ESWT is ineffective for treating non-calcific rotator cuff tendonitis (51, 54, 55). Based on the results of the subgroup analysis, we obtained a new conclusion: that low-energy ESWT (EFD < 0.12 mj/mm^2^) did not relieve pain in patients with calcific and non-calcific rotator cuff tendonitis but was effective in improving function in both.

Currently, most ESWT for tendonitis is categorized as “focal ESWT” (FESWT). Some researchers have focused on exploring the efficacy of radial or focal ESWT in tendonitis. According to previous studies, both FESWT and RESWT are effective in relieving pain in patients with tendonitis (18, 56). The mechanism of action is that in addition to its direct analgesic and anti-inflammatory effects, ESWT also induces long-term tissue regeneration, and its main biological effects on tissues are accelerated metabolism of inflammatory mediators, promotion of neovascularization, and inhibition of pain nerve signaling (57). Between these two different treatments, FESWT is more intense within a targeted area, while RESWT has a more widespread but superficial region of action (58). In addition, most tendonitis inflammation occurs at a shallower and more extensive site. Therefore, RESWT is considered a less invasive tool and is more appropriate for conservative therapy (59). Importantly, one study revealed potential anti-inflammatory protein targets of RESWT in a TNF α-induced model of acute inflammation in primary human tendon cells by quantitative proteomics, providing important insights into the molecular mechanisms underlying the anti-inflammatory role of RESWT in tendonitis. RESWT, as a new treatment in the field of rehabilitation medicine in recent years, has not only has a short treatment time and a long treatment interval but also a broad indication (60). Compared with FESWT, RESWT has better prospects for clinical treatment of tendonitis. Based on the results of our subgroup analysis, RESWT was more effective in relieving pain in upper limb tendonitis, which provides valid evidence for the clinical use of RESWT to relieve tendonitis.

In addition, to reduce heterogeneity in subgroup analyses, we also compared the efficacy of low-energy ESWT on upper limb tendonitis, include rotator cuff tendonitis, lateral epicondylitis and trigger finger. According to the results of subgroup analysis, low-energy ESWT is more effective for lateral epicondylitis. However, we were unable to include long bicipital tendonitis for subgroup analysis because the treatment modality regarding the treatment of long bicipital tendonitis was RESWT, which was not in accordance with our pre-specified principles.

Our study had several limitations. First, there were few studies on finger tendonitis and long bicipital tendonitis, and the level of evidence was weak. Second, GS tests were used to assess functional activity (loss of GS) in patients with lateral epicondylitis; the results of the assessment are not sufficient to reflect the improvement of the patient's activity function and emphasize more on the restoration of muscle strength without pain. Third, the issue of calcification that occurs when ESWT is applied to calcific rotator cuff tendonitis has not yet been explored. Fourth, some studies lacked intervention parameters such as number of pulses, energy and sessions. Although most of the studies specified the treatment sessions, subgroup analyses could not be performed owing to the duration of follow-up and energy parameters. Fifth, the shockwave devices result in different physical effects, and there are differences in treatment areas and treatment modalities. Finally, differences in ESWT instruments, literature sources, and intervention parameters between studies were also important factors influencing the trial results, which increased their heterogeneity. However, the current number of studies and trial data are insufficient to exclude these factors using subgroup analysis. Therefore, more multicentre, follow-up, double-blind, RCT trials should be conducted in the future to explore optimal treatment options to improve the clinical efficacy of ESWT for tendonitis.

## 5 Conclusion

In conclusion, compared with placebo, high-energy ESWT was effective in improving pain and function in patients with upper extremity tendonitis, especially RESWT. Therefore, ESWT may have an important role to play as an effective physical therapy for the treatment of pain and functional limitations induced by tissue damage.

## Data availability statement

The raw data supporting the conclusions of this article will be made available by the authors, without undue reservation.

## Author contributions

YX: Data curation, Software, Validation, Writing – original draft, Writing – review & editing. TW: Data curation, Writing – original draft. SJ: Methodology, Software, Validation, Writing – review & editing. LL: Conceptualization, Data curation, Investigation, Writing – review & editing. QS: Software, Validation, Writing – review & editing. YP: Investigation, Methodology, Writing – review & editing. QZ: Data curation, Project administration, Writing – review & editing. WL: Supervision, Writing – original draft, Writing – review & editing.
